# Comparison of the amniotic fluid and fetal urine peptidome for biomarker discovery in renal developmental disease

**DOI:** 10.1038/s41598-020-78730-3

**Published:** 2020-12-10

**Authors:** Camille Fédou, Benjamin Breuil, Igor Golovko, Stéphane Decramer, Pedro Magalhães, Françoise Muller, Sophie Dreux, Petra Zürbig, Julie Klein, Joost P. Schanstra, Bénédicte Buffin-Meyer

**Affiliations:** 1 Institut National de La Santé Et de La Recherche Médicale (INSERM), U1048/I2MC-Equipe 12, Institut of Cardiovascular and Metabolic Disease, 1 Avenue Jean Poulhès, BP 84225, 31432 Toulouse Cedex 4, France; 2 Université Toulouse III Paul-Sabatier, Toulouse, France; 3 Mosaiques Diagnostics GmbH, Hannover, Germany; 4 Hôpital Des Enfants, Service de Néphrologie - Médecine Interne - Hypertension Pédiatrique, CHU Toulouse, Toulouse, France; 5 Unité de Biochimie Fœto-Placentaire, Laboratoire de Biochimie - Hormonologie CHU Robert Debré, AP-HP, Paris, France

**Keywords:** Prognostic markers, Paediatrics, Kidney diseases, Paediatric research

## Abstract

Production of amniotic fluid (AF) is view as predominately driven by excretion of fetal urine (FU). However, the origin of AF peptides, often considered as potential biomarkers of developmental diseases, has never been investigated. Here, we evaluated the FU origin of AF peptides and if the AF peptide content can be used as a surrogate of FU. The abundance of endogenous peptides was analyzed by capillary electrophoresis coupled to mass spectrometry in 216 AF and 64 FU samples. A total of 2668 and 3257 peptides was found in AF and FU respectively. The AF peptidome largely overlapped with the FU peptidome, ranging from 54% in the second pregnancy trimester to 65% in the third trimester. Examination of a subset of 16 paired AF and FU samples revealed that 67 peptides displayed a significant positively correlated abundance in AF and FU, strongly suggesting that their presence in AF was directly associated to FU excretion. As proof-of-concept we showed that measuring the AF abundance of these 67 peptides of FU origin allowed prediction of postnatal renal survival in fetuses with posterior urethral valves. These results demonstrate that the AF peptidome can be considered as a good surrogate of the FU peptidome.

## Introduction

Exploring the urinary peptidome (“peptidomics”) in both adults and children led to the identification of peptide-based signatures allowing diagnosis/prognosis of diseases including kidney^1–4^ or cardiovascular diseases^5–7^, as well as cancers^8,9^. Peptidomics has also been successfully applied in the prenatal context. We identified and validated 12 peptides in fetal urine (FU) that predicted in utero postnatal renal survival in fetuses with developmental renal disease (*i.e.* posterior urethral valves, PUV)^10,11^. However, in contrast to postnatal urine, sampling of FU is invasive since it is collected directly from the fetal bladder. Amniotic fluid (AF) can also be used as a reliable source of biomarkers for fetal diseases^12^. We found a panel of 34 AF peptides that predicted the neurological severity of congenital cytomegalovirus (CMV) infection^13^. More recently, using a prospective multicentre cohort including 178 patients, we identified an AF signature containing 98 peptides that predicted with high accuracy the postnatal renal outcome in fetuses having bilateral kidney malformations (CAKUT for congenital anomalies of the kidney and urinary tract), outperforming predictions based on currently used clinical methods^14^. The presence of such markers in AF is thus particularly interesting since AF sampling is less invasive than FU collection.

AF is depending on both fetal and maternal tissues. During the first half of the pregnancy, AF is the transudate of plasma from the fetus across the not-yet-keratinized fetal skin or from the mother across the placenta surface. In the second half of the pregnancy, when the fetal skin is fully keratinized, AF production results from fetal urine excretion and fetal pulmonary fluid secretion whereas AF removal is mainly driven by fetal swallowing and transfer to the fetal circulation across the amniotic membrane^15,16^. Comparison of the protein content of AF and (adult) urine showed that 73% of proteins present in AF were also detected in urine^17^. However, no data is available regarding the overlap between FU and AF peptides with the aim to determine whether AF peptides could be used as a surrogate for FU peptides.

Here we investigated the origin of AF peptides by performing a comprehensive analysis of AF and FU peptidome depending on gestational age. A total of 280 AF and FU samples analysed for peptide profiles using capillary electrophoresis coupled to mass spectrometry (CE-MS) were compared. The overlap of peptides present in these two body fluids together with their correlation in abundance were evaluated. Finally, as a proof-of-concept, the potential of quantifying the abundance of AF peptides of FU origin for predicting fetal renal disease progression was assessed.

## Results

Two hundred eighty fetuses with gestational age extending from 11 to 39 weeks of amenorrhea (WA) (Fig. S1A) were included from retrospective CE-MS-based peptidomics studies^10,13,14,18^. For AF analysis, 216 patients were included (Fig. S1B): thirteen fetuses were healthy, 47 were CMV infected and 156 had CAKUT. The major CAKUT aetiology was lower urinary tract obstruction mainly associated with PUV (45/156, Table S1). For FU analysis, 64 patients were included (Fig. S1C): all were CAKUT fetuses with PUV. For a subset of 16 patients, paired FU and AF samples were available.

### AF and FU peptide content

**Figure 1:**
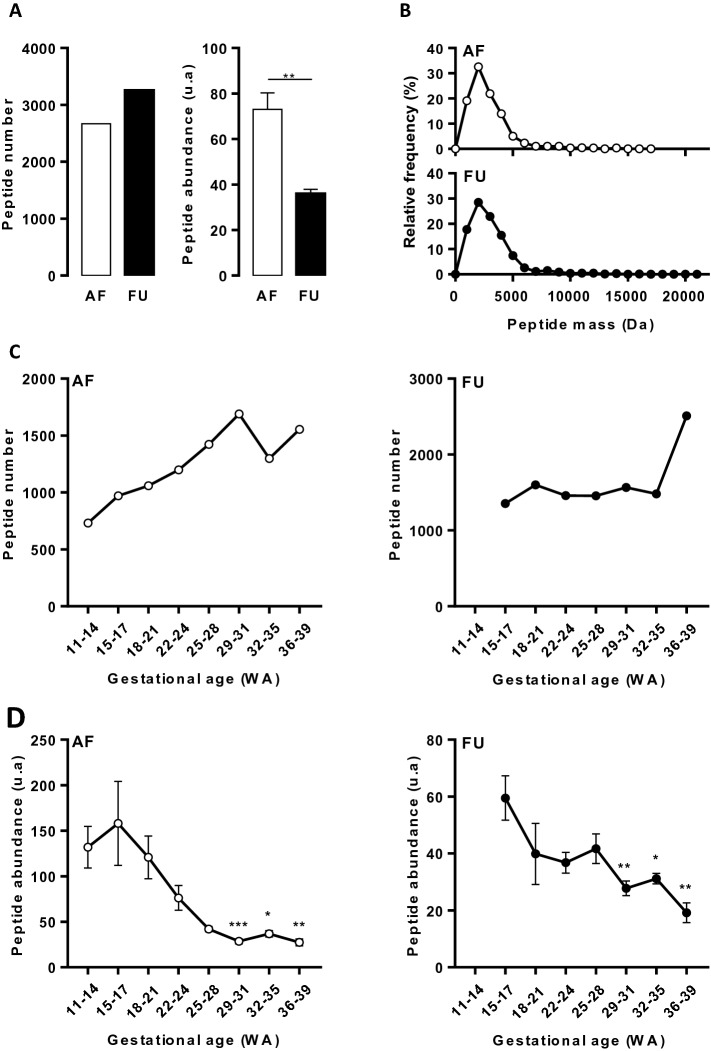
Amniotic fluid and fetal urine peptidome and evolution with gestational age. (**A**) Number and abundance of peptides in amniotic fluid (AF) and fetal urine (FU) irrespective of gestational age. (**B**) Weight distribution of peptides present in AF and FU. The mean and the median were respectively 2945 Da and 2425 Da for AF peptidome and 3110 Da and 2647 Da for FU peptidome. (**C**) Gestational age evolution of peptide number in AF (left panel) and FU (right panel). (**D**) Gestational age evolution of peptide abundance in AF (left panel) and FU (right panel). Data are means ± SEM. *p < 0.05, **p < 0.01, ***p < 0.001 *versus* 11–14 WA (AF) or 15–17 WA (FU), according to Mann–Whitney test (A) or One-way Anova (D).

A total of 2668 peptides were detected in AF whereas 3257 peptides were found in FU (Fig. 1A). Despite this lower molecular diversity, average abundance of peptides in AF was higher than in FU (Fig. 1A). In both body fluids, 90% of the detected peptides had a mass comprised between 800 and 5000 Da (Fig. 1B). However, the molecular mass of the peptides was slightly lower in AF (means ± SEM: 2945 + /- 38 Da in AF *versus* 3110 + /- 36 Da in FU; p < 0.0001). Sequence information was available for 607 and 620 peptides in AF and FU, respectively. The majority of peptides originated from various collagens (~ 75%). The other most frequently found peptides included fragments of insulin-like growth factor with associated binding protein (~ 4%) or fibrinogen (~ 3%) followed by peptides derived from osteopontin, apolipoprotein, hemoglobin or inter-alpha-trypsin inhibitor heavy chain H4. The most abundant AF peptides (top 2.5%) included 11 fragments of collagen (together covering 4% of the total detected peptide signal), 4 fragments of thymosin beta 4 (1% of total signal), 1 cornulin peptide and 1 heparin cofactor 2 fragment. In contrast, the most abundant FU peptides (top 2.5%) were all derived from collagen and explained 12% of the total peptide intensity. The number of peptides in AF increased with gestational age until 29–31 WA and then stabilized whereas in FU no increase in the number of peptides was observed, except in late pregnancy (Fig. 1C). In both body fluids, a reduction of peptide abundance was observed with increasing gestational age (Fig. 1D).

### AF and FU peptidome overlap

**Figure 2:**
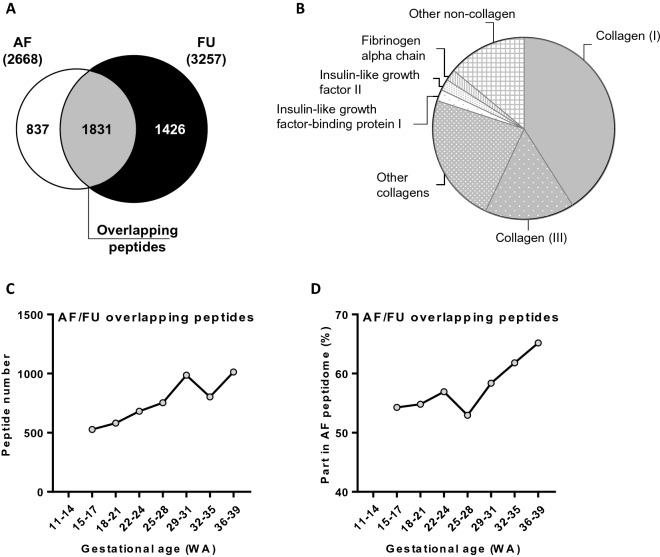
Overlap in the amniotic fluid and fetal urine peptidome. (**A**) Overlapping peptides in both amniotic fluid (AF) and fetal urine (FU), irrespective of gestational age. The total number of peptides identified is indicated in parentheses. (**B**) Protein origin of common peptides. (**C**) Gestational age evolution in the number of overlapping peptides (left) and percentage represented in the AF peptide content (right). (**D**) Gestational age evolution in the abundance in AF of overlapping peptides (left) and its percentage represented in the AF peptide abundance (right).

Analysis of the overlap in AF and FU peptidome over the full gestational period led to the identification of 1831 peptides detectable in both AF and FU (Fig. 2A) representing 69% of the total number and 72% of total abundance of AF peptides. This suggests that an important fraction of AF peptides might originate from FU. The majority of common sequenced peptides were various collagens (383 peptides) or other proteins including fibrinogen (10 peptides), insulin-like growth factor (8 peptides) and insulin-like growth factor-binding protein (8 peptides) (Fig. 2B). The number of common peptides increased with gestational age, and the part of AF peptidome which overlapped with FU peptidome increased from 54 to 65% between the second and the third pregnancy trimester (Fig. 2C,D).

### Correlation of overlapping peptides in AF and FU

**Figure 3:**
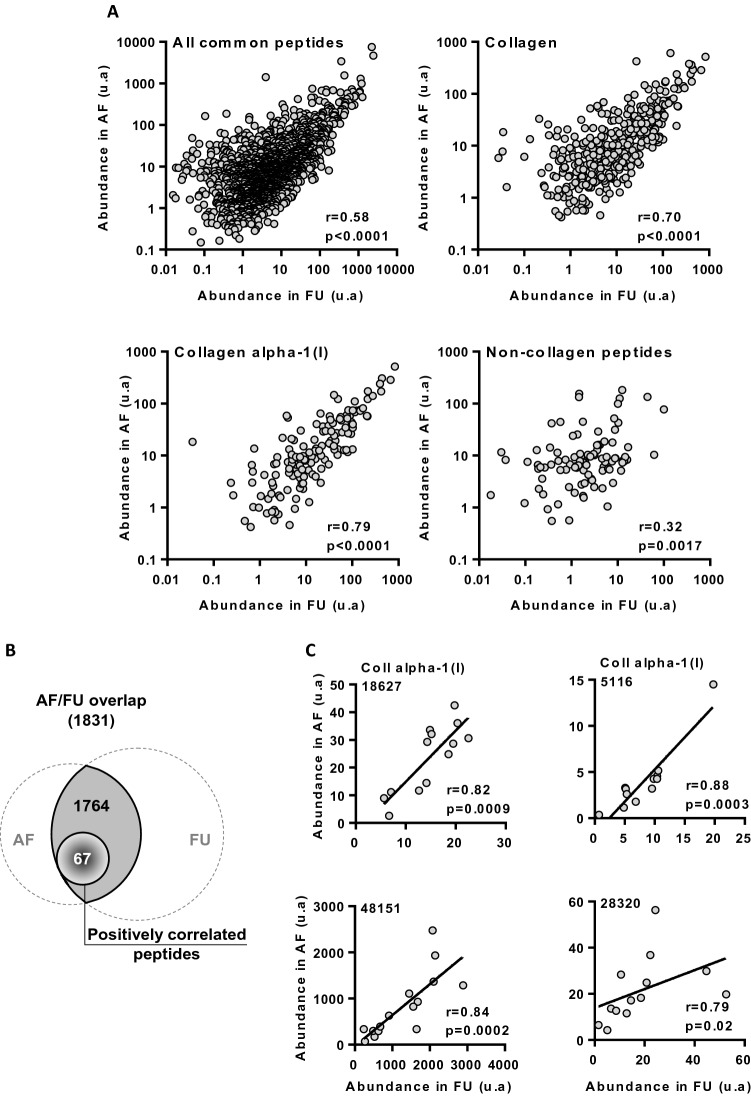
Analysis of matched amniotic fluid and fetal urine samples. (**A**) Correlation of the average abundance in AF of all, collagen, collagen alpha-1(I) and non-collagen overlapping peptides with their average abundance in FU irrespective of gestational age. r, Spearman correlation coefficient. (**B**) Number of common peptides whose abundance is correlated in matched amniotic fluid (AF) and fetal urine (FU) from a subset of 16 fetuses. The total number of peptides in the overlap is indicated in parentheses. (**C**) Correlation between abundance of 4 representative overlapping peptides. r, Spearman correlation coefficient. 18627, fragment of collagen alpha-1(I) chain; 5116, fragment of collagen alpha-1(I) chain; 48151 and 28320, non-sequenced peptides. Coll, collagen.

A strong positive correlation between the average abundance of 1831 overlapping peptides in AF and the average abundance in FU was found (r = 0.58, p < 0.0001) (Fig. 3A), thereby indicating that common peptidome was globally correlated in the two fluids. This correlation was mainly driven to overlapping collagen fragments (r = 0.70, p < 0.0001), particularly peptides that issued from collagen alpha-1 (I) chain (r = 0.79, p < 0.0001).

Next, using the subset of 16 paired AF and FU samples, we identified 67 common peptides whose abundance in AF was significantly positively correlated to peptide abundance in FU (Fig. 3B). These AF peptides were thus considered as having predominantly a FU origin. They included different fragments of collagen (15 peptides) and osteopontin (1 peptide) (Table S2). For example, peptides 18625 and 5116 (both derived from Collagen alpha-1(I) chain), as well as peptides 48151 and 28320 displayed high correlation coefficients (Fig. 3C).

### AF biomarkers for fetal diseases

As shown here, since FU is the primary source for the AF peptidome, we hypothesized that measuring predominantly FU-derived peptides in AF could be used as a surrogate for FU peptides to prognosticate renal developmental disease, thereby reducing the invasiveness of the procedure to obtain the desired body fluid. As a proof-of-concept study, we tested this hypothesis for the prediction of postnatal renal survival in a small set of 30 fetuses with PUV (Table S3).

**Figure 4:**
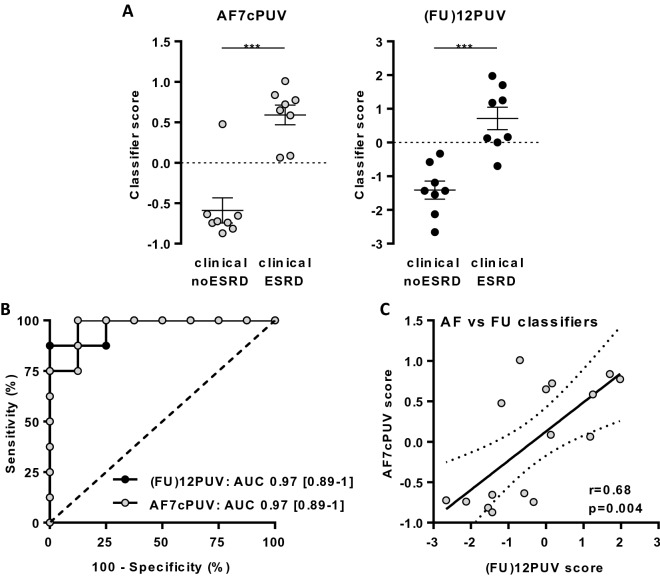
Potential of amniotic peptides for the prediction of renal survival in PUV fetuses. (**A**) Scatter dot plots showing AF7cPUV and (FU)12PUV classifier-based scores in 16 PUV patients from the validation cohort. The dotted horizontal line indicates the cutoff score (0 for both AF7cPUV and (FU)12PUV). A score > cutoff suggested early development of ESRD whereas a score < cutoff predicted fetus in the noESRD group. Data are means ± SEM. ***p < 0.001 *versus* noESRD according to Mann–Whitney test. (**B**) Compared ROC curves of AF7cPUV and (FU)12PUV. Brackets indicated confidence intervals for AUC. (**C**) Correlation between AF7cPUV and (FU)12PUV based scores.

Focusing on the 67 AF/FU common and correlated peptides in a discovery cohort of 14 PUV patients (6 with early ESRD and 8 without ESRD at 2 years postnatally (noESRD)) yielded 7 peptides with significantly different abundance in AF. The 7 peptides were included in a random forest discrimination model (called ‘AF7cPUV’) which was optimized for the separation of the two patient populations. The classifier was next validated in a test cohort of 16 other PUV fetuses (8 ESRD and 8 noESRD patients with matched AF and FU samples). This validation yielded a prediction of postnatal renal survival with 100% sensitivity [95% confidence interval CI: 63–100], 87.5% specificity [95%CI: 47–100] and an area under the receiver-operating-characteristic curve (AUC) of 0.97 [95%CI: 0.89–1] (Fig. 4A,B). The AF7cPUV based prediction was similar to the performance of a previously published classifier including 12 fetal urinary peptides (‘(FU)12PUV’; sensitivity 87.5% [95%CI: 47–100], specificity 100% [95%CI: 63–100], AUC 0.97 [95%CI: 0.89–1])^10^ (Fig. 4A,B). In addition, the AF7cPUV and 12PUV scores for these patients were significantly correlated (Fig. 4C). Thus, the current proof-of-concept study supports the use of AF in fetal disease as a surrogate of FU.

## Discussion

The present study compared the AF and FU peptide profiles of 280 fetuses with the aim to determine the origin of the AF peptidome. As main result, we observed that AF peptidome largely overlaps with FU (from 54% in the second trimester to 65% in the third pregnancy trimester), thereby suggesting that an important fraction of amniotic peptides originates, at least in part, from FU. A proof-of-concept was provided for the use of the AF peptidome as a source of surrogate biomarkers of FU origin.

To the best of our knowledge, the present report is the first that compared amniotic and fetal urinary peptidome. Therefore, data showing that > 50% peptides present in AF were also identified in FU expand the current knowledge in the field.

It has already been shown that both fetal diuresis and (adult) urinary proteins predominantly participated to AF volume^16,19^ and proteome^17^, respectively. Here, we added the urinary peptidome component as an important contributor in AF peptide content, thereby supporting the concept that AF production is strongly driven by FU excretion.

We considered that peptides detected both in FU and AF originated from FU. Nevertheless, we cannot rule out the possibility that some of these peptides derived from an additional source. AF peptides potentially also result from fetal respiratory tract secretions, recognized as another significant source of AF. Although no information on the peptide content of fetal lung fluid is available, such hypothesis has to be taken account since collagen (I) (from which most AF/FU sequenced common peptides are derived) is an abundant matrix component in the developing lung^20,21^. In contrast, for the 67 peptides displaying a significant positive correlation of abundance in AF and FU, any origin other than urine can reasonably be excluded.

Almost half of FU peptides (1426 peptides) appear to be specific for FU since undetectable in AF (Fig. 2A). These peptides were mainly (in number and abundance) derived from the different collagen chains. They were also less abundant in urine than the AF/FU overlapping peptides (Fig. S2A). Their absence in AF may seem surprising at first glance since the urinary tract opens into the amniotic cavity. However, they might be present in AF but diluted below the detection limits (Fig. S2A). Moreover, some FU specific peptides can be digested by peptidases present downstream the bladder from where FU samples were collected. To evaluate this hypothesis, we performed a simulation using the ExPaSy software (https://web.expasy.org/peptide_cutter/)^22^. Results showed that 19 out of 22 fragments of Collagen alpha-1(I) chain exclusively found in FU could be degraded by enzymes potentially present in AF such pepsin, trypsin, chymotrypsin and Arg-C proteinase^23,24^ (Table S4).

A total of 837 peptides was exclusively present in AF. These included peptides of collagens (69 peptides), apolipoprotein (5 peptides), complement (5 peptides) and inter-alpha-trypsin inhibitor heavy chain H4 (5 peptides). One fragment of heparin cofactor 2, 4 fragments of thymosin beta 4, and 1 cornulin sequence were among the most abundant specific AF peptides. It is unlikely that these peptides would have escaped detection in the urine due to low abundance since their abundance in AF was similar to that of AF/FU common peptides (Fig. S2B). However, a number of these peptides could derive from other fetal sources or from maternal circulation instead of FU. They could also result from the digestion of urinary proteins, as demonstrated by an analysis of the cleavage site specificity performed using Proteasix algorithm (http://proteasix.cs.man.ac.uk/)^25^ that yielded a list of 27 potential AF proteases possibly liable for the generation of 129 AF restricted sequenced peptides (Table S5).

A recent study has proposed that the majority of urinary peptides may originate from the kidney^26^. By extension, our results suggest that the AF peptidome may represent to a certain extent processes occurring in the developing kidney. This hypothesis is supported by our proof-of-concept study that shows that focusing on the AF abundance of 67 AF/FU correlated common peptides allowed prediction of postnatal renal survival in fetuses with posterior urethral valves. We recently started the ANTENATAL study (NCT03116217^27^), an international, prospective and case–control study aiming to recruit more than 400 fetuses with PUV over a range of 4 years for the multicenter validation of the initially identified FU peptide-based classifier^10^. The ANTENATAL study will be an excellent opportunity to reevaluate in a much larger dataset the potential of focusing on urine-derived peptides in AF for diagnosis of fetal renal disease.

Other studies have explored the AF content and origin. However, while most studies were based on a limited set of fetuses^28–31^, we have here collected an important number of both AF and FU samples (216 and 64, respectively). This high sample number ranging throughout second and third trimester of pregnancy also allowed a precise vision of gestational age-dependent changes in AF peptide content. Moreover, while a previous study investigated the urinary origin of AF proteins comparing AF to adult urine^17^, in this study we compared for the first time AF to FU, allowing a much more physiological comparison of the urine and AF content. Finally, a subset of matched AF and FU samples was available in which the peptidome was analyzed under the same operating conditions, within the same laboratory, with the same CE-MS device, by a unique operator and over a short interval of time, thereby allowing a robust comparison.

One limitation of this study is that both AF and FU samples were mainly collected from pathological (mainly CAKUT) pregnancies, potentially hampering the generalization of the conclusions. Moreover, we were not able to explore the AF peptidome at the beginning of the pregnancy as amniocentesis is rarely done before the end of the first trimester. However, it is expected that the AF composition is similar to that of fetal plasma during this period which contains potentially less biomarkers of fetal kidney development. Finally, only 23% and 19% of the AF and FU peptides were sequenced, respectively. Additional sequence analysis could shed further light on the overlap.

In conclusion, these results suggest that AF peptidome can be considered as a good surrogate of the FU peptidome and thus represent a potential source of biomarkers for developmental kidney disease.

## Methods

### Patients

The present study is a retrospective analysis of data previously reported by our laboratory^10,13,14^. It included 13 healthy fetuses^14^, 47 fetuses with congenital CMV infection^13^ and 220 fetuses with CAKUT^10,14^ recruited in multidisciplinary prenatal diagnosis centers in France and Belgium. Based on the distribution of available samples the gestational period was divided in eight periods: 11–14 WA, 15–17 WA, 18–21 WA, 22–24 WA, 25–28 WA, 29–31 WA, 32–35 WA and 36–39 WA (Fig. S1).

In France, research based on retrospective data is excluded from the law relating to research involving the human subjects (known as the Jardé law) because it does not involve individuals but data. In this case, ethical opinion is not required. The present study related exclusively to the re-use of data already collected and published (available as supplementary data^10,13,14^) and required in no way access to the identifiable patient data since publicly available in the publications. Therefore, it does not fall into the category of research covered by the Jardé law as mentioned above. For this reason, we did not seek ethics approval.

### Peptidomics data

Details for sample collection and preparation, CE-MS-based peptidome analysis and associated data processing, as well as peptide sequencing, have been previously described in the original publications^10,13,14^ and can be found in the Supplementary methods. In the present study, only peptides that were detected in one biological fluid (AF or FU) with a minimum frequency of 50% in at least one gestational period were investigated.

### AF peptide based classifier

The potential of AF peptides to prognosticate the severity of developmental renal disease was evaluated in a subset of 30 fetuses with PUV. The judgment criterion was the development—or not—of ESRD at 2 years postnatally^10,18^. The ESRD group included PUV patients (14/30) that died in the neonatal period due to ESRD or that were subjected to TOP due to the severity of renal lesions. The noESRD group was composed of PUV patients (16/30) that led to liveborn infants having glomerular filtration rate (estimated using Schwartz formula) > 15 ml/min at 2 years of life. Fetuses were divided into independent discovery and validation subsets for generation and subsequent testing of the omics-based prognosis classifier, respectively. Significant AF peptides of FU origin were modelled using an in-house developed tool^32^ into a Random Forest (RF)-based classifier (RF package of R^33^) to generate the prognostic AF7cPUV classifier. The classifier was optimized for the prognosis of the 14 patients of the training set. The cutoff was determined as the score which allowed the optimal discrimination of PUV patients with early ERSD from PUV patients without postnatal ESRD. The number of trees was fixed to 1000 and the other parameters were kept as default values. Predictive performance was assessed by calculating sensitivity, specificity and AUC using MedCalc (version 16.8, MedCalc Software). Two-sided confidence 95% CI were calculated to quantify the statistical precision of the measurements.

### Statistical analyses

Comparison of peptide abundances, molecular masses and classifier-based scores of PUV fetuses were performed using a Mann–Whitney test. Effect of gestational age on peptide abundance was assessed according to One-way Anova. Relation between peptide abundances in AF and FU was evaluated performing Spearman correlation test. Peptides with significantly different AF abundance in PUV patients with early ESRD compared to PUV fetuses without ESRD at 2 years postnatally were selected by Wilcoxon analysis. To assess the discriminatory ability of the classifier, the hypothesis that the AUC is 0.5 (value indicating "no discrimination") was tested^34^. In all cases, two-sided tests were conducted and p < 0.05 was considered as statistically significant.

## Supplementary information

Supplementary Information 1.
