# Supplementary material for: Comparison of the amniotic fluid and fetal urine peptidome for biomarker discovery in renal developmental disease
Source: Sci Rep. 2020 Dec 10;10:21706. doi: 10.1038/s41598-020-78730-3 (PMC7729974; doi:10.1038/s41598-020-78730-3)
Supplement: Supplementary file 1 — Supplementary Information 1. [file 41598_2020_78730_MOESM1_ESM.pdf]

Supplementary material

Comparison of the amniotic fluid and fetal urine peptidome for biomarker discovery in renal developmental disease

Camille Fedou, Benjamin Breuil, Igor Golovko, Stéphane Decramer, Pedro Magalhães, Françoise Muller, Sophie Dreux, Petra Zürbig, Julie Klein, Joost P Schanstra, Bénédicte Buffin-Meyer

Contents:

Supplementary Methods
Supplementary Figure 1
Supplementary Figure 2
Supplementary Table 1
Supplementary Table 2
Supplementary Table 3
Supplementary Table 4
Supplementary Table 5

Supplementary Methods

Sample collection

Amniotic fluid (AF) was collected according to local management under ultrasound guidance and frozen at -20°C locally. In case of anhydramnios, samples were obtained after amnioinfusion. Fetal urine (FU) was collected from the bladder of fetuses under ultrasound guidance and frozen at -20°C locally. Samples were then shipped on dry ice to the central laboratory (Inserm U1048, Toulouse, France).

Peptidome analysis

Sample preparation. Briefly, immediately before preparation, AF and FU samples kept at –80°C were thawed and 150 µl aliquots were diluted with the same volume of 2 M urea, 10 mM NH4OH containing 0.2% SDS. Subsequently, samples were passed over a Centristat 20-kDa cut-off centrifugal filter device (Sartorius) in order to eliminate high molecular weight compounds. The filtrate was desalted using a NAP-5 gel filtration column (GE Healthcare) to remove urea and electrolytes. Lyophilisation of the samples was performed using a Savant speedvac SVC100H connected to a Virtis 3L Sentry freeze dryer (Fisher Scientific) and stored at 4°C until use.

CE-MS analysis. Shortly before CE-MS analysis, the samples were re-suspended in 10µL of HPLC grade H2O. CE-MS analyses were performed using a Beckman Coulter Proteome Lab PA800 capillary electrophoresis system (Beckman Coulter) on-line coupled to a micrOTOF II MS (Bruker Daltonic). The electro-ionization sprayer (Agilent Technologies) was grounded, and the ion spray interface potential was set between –4 and –4.5 kV. Data and MS acquisition methods were automatically controlled by the CE via contact-close-relays. Spectra were accumulated every 3 s, over a range of m/z 350 to 3000.

Data processing. Mass spectral ion peaks representing identical molecules at different charge states were deconvoluted into single masses using MosaiquesVisu software[1]. The software automatically examined all mass spectra from a CE-MS analysis for signals with a signal-to-noise ratio at least four present in three consecutive spectra. Furthermore, the isotopic distribution was assessed, and charge was assigned based on the isotopic distribution, as well as conjugated masses, using a probabilistic clustering algorithm. This operation resulted in a list wherein all signals that could be interpreted are defined by mass/charge, charge, migration time, and signal intensity (ion counts). Time-of-flight mass spectrometry (TOF-MS) data were calibrated utilizing Fourier transform ion cyclotron resonance mass spectrometry (FT-ICR-MS) data as reference masses applying linear regression. Normalization of the amplitude of the peptides was based on sequenced endogenous "housekeeping" peptides that varied little among the samples.

Sequencing of peptides

Native peptides from fetal urine and amniotic fluid were sequenced using LC-MS/MS and CE-MS/MS analysis[2]. LC-MS/MS analysis experiments were performed on a Dionex Ultimate 3000

RSLC nano flow system (Dionex, Camberly UK). For CE-MS/MS, the samples were injected under constant flow and pressure conditions at a pH of 2.2 to ensure that all peptides are positively charged. Both, CE and LC, were directly interfaced with an LTQ-Orbitrap XL (Thermo Finnigan, Bremen, Germany), using data-dependent HCD MS/MS sequencing of a maximum of the top 20 ions. All resultant MS/MS data were analyzed using Proteome Discoverer 1.2 (activation type: HCD; min-max precursor mass: 790–6000; precursor mass tolerance: 10 ppm; fragment mass tolerance: 0.05 Da; S/N threshold: 1) and were searched against the Uniprot human non-redundant database without enzyme specificity. No fixed modifications were selected, oxidation of methionine and proline and deamidation of aspartic acid and glutamine were selected as variable modifications. The peptide data were extracted using high confidence peptides, defined by an Xcorr≥1.9, a delta mass between experimental and theoretical mass ± 5 ppm, absence of cysteine in the sequence as without reduction and alkylation it is forms disulphide bonds, absence of oxidized proline in protein precursors other than collagens or elastin, and top one peptide rank filters.

For further validation of peptide identification, the strict correlation between peptide charge at pH 2 and CE-migration time was utilized to minimize false-positive identification rates[3]. Calculated CE-migration time of the sequence candidate based on its peptide sequence (number of basic amino acids) was compared to the experimental migration time. Peptides were accepted only if they had a mass deviation below ±50 ppm and a CE-migration time deviations below ±2 min.

References

1 Neuhoff, N. *et al.* Mass spectrometry for the detection of differentially expressed proteins: a comparison of surface-enhanced laser desorption/ionization and capillary electrophoresis/mass spectrometry. *Rapid communications in mass spectrometry : RCM* **18**, 149-156, doi:10.1002/rcm.1294 (2004).

2 Klein, J., Papadopoulos, T., Mischak, H. & Mullen, W. Comparison of CE-MS/MS and LC-MS/MS sequencing demonstrates significant complementarity in natural peptide identification in human urine. *Electrophoresis* **35**, 1060-1064, doi:10.1002/elps.201300327 (2014).

3 Zurbig, P. *et al.* Biomarker discovery by CE-MS enables sequence analysis via MS/MS with platform-independent separation. *Electrophoresis* **27**, 2111-2125, doi:10.1002/elps.200500827 (2006).

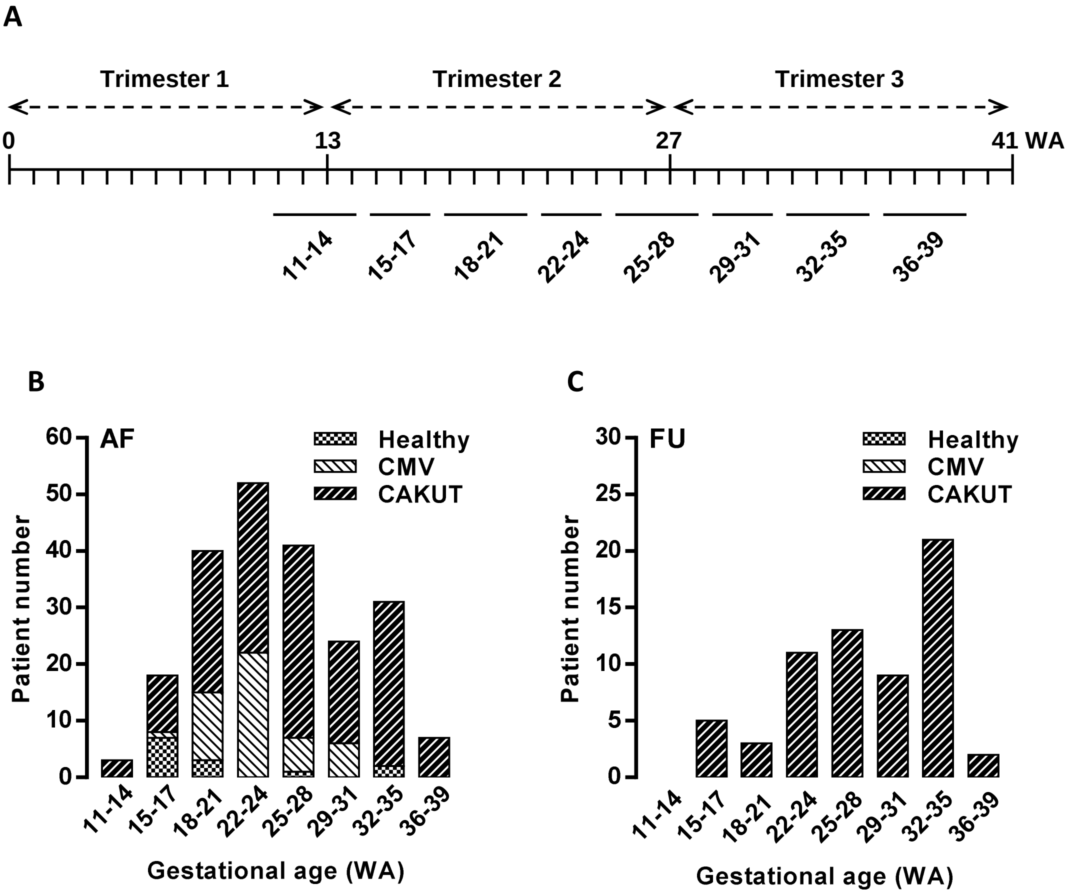

Figure S1: Distribution of patients by gestational age and disease.

A- Based on the distribution of available samples the gestational period was divided in eight periods: 11-14 weeks of amenorrhea (WA), 15-17 WA, 18-21 WA, 22-24 WA, 25-28 WA, 29-31 WA, 32-35 WA and 36-39 WA. **B and C-** Number of patients included for amniotic fluid (AF, **B**) and fetal urine (FU, **C**) peptidome studies. Healthy, pregnancies tested for chromosomal abnormalities but that were found normal; CMV, fetuses with congenital cytomegalovirus infection; CAKUT, patients with bilateral congenital anomalies of the kidney and urinary tract.

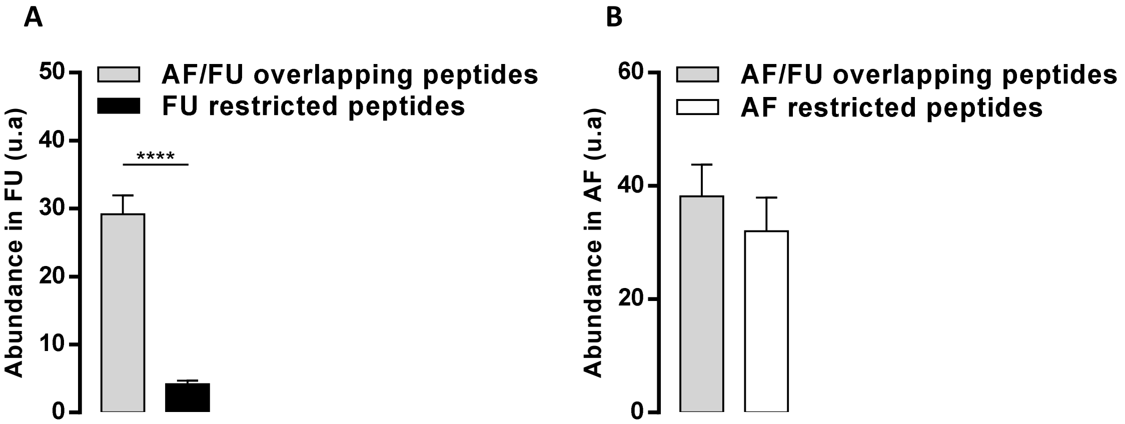

Figure S2: Abundance in FU and AF of FU- or AF restricted peptides, respectively.

A- Abundance of AF/FU overlapping peptides and FU restricted peptides in fetal urine (FU). **B-** Abundance of AF/FU overlapping peptides and AF restricted peptides in amniotic fluid (AF). Data are means ± SEM. ****p<0.0001 *versus* AF/FU overlapping peptides, according to Student test.

Supplementary Table 1: Characteristics of the study population

Patient code	Analysed biological fluid	Origin of raw data (PMID)	Group	Antenatal etiology (if available)	Amniotic fluid volume "	Gestational age (in weeks of amenorrhea)	Gestational age (in 1/4 trimester)
1	AF	Klein *et al* , Kidney International, *in press*	Healthy		Normal	20	6
2	AF	Klein *et al* , Kidney International, *in press*	Healthy		Normal	19	6
3	AF	Klein *et al* , Kidney International, *in press*	Healthy		Normal	16	5
4	AF	Klein *et al* , Kidney International, *in press*	Healthy		Normal	33	10
5	AF	Klein *et al* , Kidney International, *in press*	Healthy		Normal	16	5
6	AF	Klein *et al* , Kidney International, *in press*	Healthy		Normal	32	10
7	AF	Klein *et al* , Kidney International, *in press*	Healthy		Normal	26	8
8	AF	Klein *et al* , Kidney International, *in press*	Healthy		Normal	16	5
9	AF	Klein *et al* , Kidney International, *in press*	Healthy		Normal	16	5
10	AF	Klein *et al* , Kidney International, *in press*	Healthy		Normal	16	5
11	AF	Klein *et al* , Kidney International, *in press*	Healthy		Normal	17	5
12	AF	Klein *et al* , Kidney International, *in press*	Healthy		Normal	18	6
13	AF	Klein *et al* , Kidney International, *in press*	Healthy		Normal	17	5
14	AF	26808779	CMV		Normal	17	5
15	AF	26808779	CMV		Normal	19	6
16	AF	26808779	CMV		Normal	20	6
17	AF	26808779	CMV		n.a	20	6
18	AF	26808779	CMV		Normal	19	6
19	AF	26808779	CMV		Normal	20	6
20	AF	26808779	CMV		n.a	20	6
21	AF	26808779	CMV		Normal	20	6
22	AF	26808779	CMV		n.a	18	6
23	AF	26808779	CMV		Normal	20	6
24	AF	26808779	CMV		Normal	21	6
25	AF	26808779	CMV		Normal	20	6
26	AF	26808779	CMV		Normal	20	6
27	AF	26808779	CMV		n.a	22	7
28	AF	26808779	CMV		Normal	23	7
29	AF	26808779	CMV		n.a	23	7
30	AF	26808779	CMV		n.a	22	7
31	AF	26808779	CMV		n.a	22	7
32	AF	26808779	CMV		n.a	23	7
33	AF	26808779	CMV		n.a	24	7
34	AF	26808779	CMV		n.a	22	7
35	AF	26808779	CMV		n.a	24	7
36	AF	26808779	CMV		Normal	24	7
37	AF	26808779	CMV		n.a	23	7
38	AF	26808779	CMV		Normal	22	7
39	AF	26808779	CMV		n.a	23	7
40	AF	26808779	CMV		n.a	23	7
41	AF	26808779	CMV		n.a	23	7
42	AF	26808779	CMV		n.a	23	7
43	AF	26808779	CMV		Normal	24	7
44	AF	26808779	CMV		Normal	24	7
45	AF	26808779	CMV		n.a	23	7
46	AF	26808779	CMV		n.a	24	7
47	AF	26808779	CMV		Normal	22	7
48	AF	26808779	CMV		Normal	23	7
49	AF	26808779	CMV		Normal	28	8
50	AF	26808779	CMV		n.a	28	8
51	AF	26808779	CMV		n.a	27	8
52	AF	26808779	CMV		Normal	27	8
53	AF	26808779	CMV		n.a	25	8
54	AF	26808779	CMV		n.a	28	8
55	AF	26808779	CMV		Normal	29	9
56	AF	26808779	CMV		Normal	29	9
57	AF	26808779	CMV		Normal	29	9
58	AF	26808779	CMV		Normal	30	9
59	AF	26808779	CMV		Normal	29	9
60	AF	26808779	CMV		Normal	29	9
61	AF	Klein *et al* , Kidney International, *in press*	Cakut	One hypoplastic and one dysplastic kidney	Oligohydramnios	30	9
62	AF	Klein *et al* , Kidney International, *in press*	Cakut	One hypoplastic and one dysplastic kidney	Normal	39	11
63	AF	Klein *et al* , Kidney International, *in press*	Cakut	One hypoplastic and one dysplastic kidney	Oligohydramnios	32	10
64	AF	Klein *et al* , Kidney International, *in press*	Cakut	Bilateral dysplasia	Oligohydramnios	34	10
65	AF	Klein *et al* , Kidney International, *in press*	Cakut	Bilateral dysplasia	Normal	33	10
66	AF	Klein *et al* , Kidney International, *in press*	Cakut	Bilateral dysplasia	n.a	19	6
67	AF	Klein *et al* , Kidney International, *in press*	Cakut	Bilateral dysplasia	Normal	26	8
68	AF	Klein *et al* , Kidney International, *in press*	Cakut	Bilateral dysplasia	Normal	32	10
69	AF	Klein *et al* , Kidney International, *in press*	Cakut	Non obstructive urinary tract anomalies	Normal	19	6
70	AF	Klein *et al* , Kidney International, *in press*	Cakut	Non obstructive urinary tract anomalies	Normal	18	6
71	AF	Klein *et al* , Kidney International, *in press*	Cakut	Non obstructive urinary tract anomalies	Normal	32	10
72	AF	Klein *et al* , Kidney International, *in press*	Cakut	Non obstructive urinary tract anomalies	Normal	22	7
73	AF	Klein *et al* , Kidney International, *in press*	Cakut	Non obstructive urinary tract anomalies	Oligohydramnios	31	9
74	AF	Klein *et al* , Kidney International, *in press*	Cakut	Non obstructive urinary tract anomalies	Normal	20	6
75	AF	Klein *et al* , Kidney International, *in press*	Cakut	Non obstructive urinary tract anomalies	Normal	22	7
76	AF	Klein *et al* , Kidney International, *in press*	Cakut	No functioning kidneys	Anhydramnios	15	5
77	AF	Klein *et al* , Kidney International, *in press*	Cakut	No functioning kidneys	Anhydramnios	16	5
78	AF	Klein *et al* , Kidney International, *in press*	Cakut	No functioning kidneys	Anhydramnios	25	8
79	AF	Klein *et al* , Kidney International, *in press*	Cakut	No functioning kidneys	Anhydramnios	18	6
80	AF	Klein *et al* , Kidney International, *in press*	Cakut	No functioning kidneys	Anhydramnios	19	6
81	AF	Klein *et al* , Kidney International, *in press*	Cakut	No functioning kidneys	Anhydramnios	23	7
82	AF	Klein *et al* , Kidney International, *in press*	Cakut	No functioning kidneys	Oligohydramnios	30	9
83	AF	Klein *et al* , Kidney International, *in press*	Cakut	No functioning kidneys	Anhydramnios	20	6
84	AF	Klein *et al* , Kidney International, *in press*	Cakut	Bilateral hypoplasia	n.a	24	7
85	AF	Klein *et al* , Kidney International, *in press*	Cakut	Bilateral hypoplasia	Normal	34	10
86	AF	Klein *et al* , Kidney International, *in press*	Cakut	Bilateral hypoplasia	Anhydramnios	23	7
87	AF	Klein *et al* , Kidney International, *in press*	Cakut	Bilateral hypoplasia	Normal	25	8
88	AF	Klein *et al* , Kidney International, *in press*	Cakut	Bilateral hypoplasia	Normal	32	10
89	AF	Klein *et al* , Kidney International, *in press*	Cakut	Bilateral hypoplasia	Normal	18	6
90	AF	Klein *et al* , Kidney International, *in press*	Cakut	Bilateral hypoplasia	Oligohydramnios	27	8
91	AF	Klein *et al* , Kidney International, *in press*	Cakut	Bilateral hypoplasia	Oligohydramnios	20	6
92	AF	Klein *et al* , Kidney International, *in press*	Cakut	Bilateral hypoplasia	Oligohydramnios	23	7
93	AF	Klein *et al* , Kidney International, *in press*	Cakut	Bilateral hypoplasia	Oligohydramnios	24	7
94	AF	Klein *et al* , Kidney International, *in press*	Cakut	Upper urinary tract obstruction	Normal	28	8
95	AF	Klein *et al* , Kidney International, *in press*	Cakut	Upper urinary tract obstruction	Normal	17	5
96	AF	Klein *et al* , Kidney International, *in press*	Cakut	Upper urinary tract obstruction	Normal	24	7

Patient code	Analysed biological fluid	Origin of raw data (PMID)	Group	Antenatal etiology (if available)	Amniotic fluid volume "	Gestational age (in weeks of amenorrhea)	Gestational age (in 1/4 trimester)
97	AF	Klein *et al*, Kidney International, *in press*	Cakut	Upper urinary tract obstruction	Normal	24	7
98	AF	Klein *et al*, Kidney International, *in press*	Cakut	Upper urinary tract obstruction	Normal	32	10
99	AF	Klein *et al*, Kidney International, *in press*	Cakut	Upper urinary tract obstruction	Normal	26	8
100	AF	Klein *et al*, Kidney International, *in press*	Cakut	Upper urinary tract obstruction	Normal	27	8
101	AF	Klein *et al*, Kidney International, *in press*	Cakut	Upper urinary tract obstruction	n.a	31	9
102	AF	Klein *et al*, Kidney International, *in press*	Cakut	Upper urinary tract obstruction	Oligohydramnios	16	5
103	AF	Klein *et al*, Kidney International, *in press*	Cakut	Upper urinary tract obstruction	Normal	24	7
104	AF	Klein *et al*, Kidney International, *in press*	Cakut	Upper urinary tract obstruction	n.a	20	6
105	AF	Klein *et al*, Kidney International, *in press*	Cakut	Upper urinary tract obstruction	Normal	32	10
106	AF	Klein *et al*, Kidney International, *in press*	Cakut	Upper urinary tract obstruction	Normal	30	9
107	AF	Klein *et al*, Kidney International, *in press*	Cakut	Upper urinary tract obstruction	Normal	25	8
108	AF	Klein *et al*, Kidney International, *in press*	Cakut	Upper urinary tract obstruction	Normal	25	8
109	AF	Klein *et al*, Kidney International, *in press*	Cakut	Upper urinary tract obstruction	Normal	25	8
110	AF	Klein *et al*, Kidney International, *in press*	Cakut	Upper urinary tract obstruction	Normal	21	6
111	AF	Klein *et al*, Kidney International, *in press*	Cakut	Abnormal solitary kidney	Normal	33	10
112	AF	Klein *et al*, Kidney International, *in press*	Cakut	Abnormal solitary kidney	Oligohydramnios	25	8
113	AF	Klein *et al*, Kidney International, *in press*	Cakut	Abnormal solitary kidney	n.a	32	10
114	AF	Klein *et al*, Kidney International, *in press*	Cakut	Abnormal solitary kidney	Normal	34	10
115	AF	Klein *et al*, Kidney International, *in press*	Cakut	Abnormal solitary kidney	Normal	29	9
116	AF	Klein *et al*, Kidney International, *in press*	Cakut	Abnormal solitary kidney	n.a	21	6
117	AF	Klein *et al*, Kidney International, *in press*	Cakut	Abnormal solitary kidney	Oligohydramnios	25	8
118	AF	Klein *et al*, Kidney International, *in press*	Cakut	Abnormal solitary kidney	Anhydramnios	26	8
119	AF	Klein *et al*, Kidney International, *in press*	Cakut	Abnormal solitary kidney	Normal	29	9
120	AF	Klein *et al*, Kidney International, *in press*	Cakut	Abnormal solitary kidney	n.a	22	7
121	AF	Klein *et al*, Kidney International, *in press*	Cakut	Abnormal solitary kidney	Oligohydramnios	22	7
122	AF	Klein *et al*, Kidney International, *in press*	Cakut	Abnormal solitary kidney	Normal	32	10
123	AF	Klein *et al*, Kidney International, *in press*	Cakut	Abnormal solitary kidney	Normal	30	9
124	AF	Klein *et al*, Kidney International, *in press*	Cakut	Abnormal solitary kidney	Oligohydramnios	22	7
125	AF	Klein *et al*, Kidney International, *in press*	Cakut	Abnormal solitary kidney	Normal	30	9
126	AF	Klein *et al*, Kidney International, *in press*	Cakut	Abnormal solitary kidney	Oligohydramnios	27	8
127	AF	Klein *et al*, Kidney International, *in press*	Cakut	Abnormal solitary kidney	Normal	28	8
128	AF	Klein *et al*, Kidney International, *in press*	Cakut	Abnormal solitary kidney	n.a	29	9
129	AF	Klein *et al*, Kidney International, *in press*	Cakut	Abnormal solitary kidney	Oligohydramnios	30	9
130	AF	Klein *et al*, Kidney International, *in press*	Cakut	Abnormal solitary kidney	Normal	32	10
131	AF	Klein *et al*, Kidney International, *in press*	Cakut	Abnormal solitary kidney	Normal	27	8
132	AF	Klein *et al*, Kidney International, *in press*	Cakut	Bilateral hyperechogenic kidneys	Normal	26	8
133	AF	Klein *et al*, Kidney International, *in press*	Cakut	Bilateral hyperechogenic kidneys	Normal	30	9
134	AF	Klein *et al*, Kidney International, *in press*	Cakut	Bilateral hyperechogenic kidneys	Normal	28	8
135	AF	Klein *et al*, Kidney International, *in press*	Cakut	Bilateral hyperechogenic kidneys	n.a	27	8
136	AF	Klein *et al*, Kidney International, *in press*	Cakut	Bilateral hyperechogenic kidneys	Oligohydramnios	24	7
137	AF	Klein *et al*, Kidney International, *in press*	Cakut	Bilateral hyperechogenic kidneys	Oligohydramnios	24	7
138	AF	Klein *et al*, Kidney International, *in press*	Cakut	Bilateral hyperechogenic kidneys	Normal	27	8
139	AF	Klein *et al*, Kidney International, *in press*	Cakut	Bilateral hyperechogenic kidneys	Oligohydramnios	13	4
140	AF	Klein *et al*, Kidney International, *in press*	Cakut	Bilateral hyperechogenic kidneys	Oligohydramnios	14	4
141	AF	Klein *et al*, Kidney International, *in press*	Cakut	Bilateral hyperechogenic kidneys	n.a	25	8
142	AF	Klein *et al*, Kidney International, *in press*	Cakut	Bilateral hyperechogenic kidneys	Normal	21	6
143	AF	Klein *et al*, Kidney International, *in press*	Cakut	Bilateral hyperechogenic kidneys	Normal	24	7
144	AF	Klein *et al*, Kidney International, *in press*	Cakut	Bilateral hyperechogenic kidneys	Normal	30	9
145	AF	Klein *et al*, Kidney International, *in press*	Cakut	Bilateral hyperechogenic kidneys	Normal	36	11
146	AF	Klein *et al*, Kidney International, *in press*	Cakut	Bilateral hyperechogenic kidneys	Normal	18	6
147	AF	Klein *et al*, Kidney International, *in press*	Cakut	Bilateral hyperechogenic kidneys	Normal	38	11
148	AF	Klein *et al*, Kidney International, *in press*	Cakut	Bilateral hyperechogenic kidneys	Oligohydramnios	35	10
149	AF	Klein *et al*, Kidney International, *in press*	Cakut	Bilateral hyperechogenic kidneys	n.a	30	9
150	AF	Klein *et al*, Kidney International, *in press*	Cakut	Bilateral hyperechogenic kidneys	n.a	34	10
151	AF	Klein *et al*, Kidney International, *in press*	Cakut	Bilateral hyperechogenic kidneys	n.a	34	10
152	AF	Klein *et al*, Kidney International, *in press*	Cakut	Bilateral hyperechogenic kidneys	Oligohydramnios	25	8
153	AF	Klein *et al*, Kidney International, *in press*	Cakut	Bilateral hyperechogenic kidneys	Oligohydramnios	29	9
154	AF	Klein *et al*, Kidney International, *in press*	Cakut	Bilateral hyperechogenic kidneys	Oligohydramnios	23	7
155	AF	Klein *et al*, Kidney International, *in press*	Cakut	Bilateral hyperechogenic kidneys	Oligohydramnios	28	8
156	AF	Klein *et al*, Kidney International, *in press*	Cakut	Bilateral hyperechogenic kidneys	Normal	26	8
157	AF	Klein *et al*, Kidney International, *in press*	Cakut	Bilateral hyperechogenic kidneys	Normal	27	8
158	AF	Klein *et al*, Kidney International, *in press*	Cakut	Bilateral hyperechogenic kidneys	Normal	28	8
159	AF	Klein *et al*, Kidney International, *in press*	Cakut	Bilateral hyperechogenic kidneys	Normal	33	10
160	AF	Klein *et al*, Kidney International, *in press*	Cakut	Bilateral hyperechogenic kidneys	Normal	28	8
161	AF	Klein *et al*, Kidney International, *in press*	Cakut	Bilateral hyperechogenic kidneys	Normal	34	10
162	AF	Klein *et al*, Kidney International, *in press*	Cakut	Bilateral hyperechogenic kidneys	Normal	26	8
163	AF	Klein *et al*, Kidney International, *in press*	Cakut	Bilateral hyperechogenic kidneys	Normal	22	7
164	AF	Klein *et al*, Kidney International, *in press*	Cakut	Bilateral hyperechogenic kidneys	n.a	26	8
165	AF	Klein *et al*, Kidney International, *in press*	Cakut	Bilateral hyperechogenic kidneys	Normal	27	8
166	AF	Klein *et al*, Kidney International, *in press*	Cakut	Bilateral hyperechogenic kidneys	Normal	20	6
167	AF	Klein *et al*, Kidney International, *in press*	Cakut	Bilateral hyperechogenic kidneys	Normal	37	11
168	AF	Klein *et al*, Kidney International, *in press*	Cakut	Bilateral hyperechogenic kidneys	Normal	23	7
169	AF	Klein *et al*, Kidney International, *in press*	Cakut	Bilateral hyperechogenic kidneys	Normal	23	7
170	AF	Klein *et al*, Kidney International, *in press*	Cakut	Bilateral hyperechogenic kidneys	Normal	31	9
171	AF	Klein *et al*, Kidney International, *in press*	Cakut	Bilateral hyperechogenic kidneys	Oligohydramnios	18	6
172	AF	Klein *et al*, Kidney International, *in press*	Cakut	Lower urinary tract obstruction	Oligohydramnios	34	10
173	AF	Klein *et al*, Kidney International, *in press*	Cakut	Lower urinary tract obstruction	Oligohydramnios	23	7
174	AF	Klein *et al*, Kidney International, *in press*	Cakut	Lower urinary tract obstruction	Oligohydramnios	34	10
175	AF	Klein *et al*, Kidney International, *in press*	Cakut	Lower urinary tract obstruction	Normal	19	6
176	AF	Klein *et al*, Kidney International, *in press*	Cakut	Lower urinary tract obstruction	Oligohydramnios	13	4
177	AF	Klein *et al*, Kidney International, *in press*	Cakut	Lower urinary tract obstruction	Oligohydramnios	23	7
178	AF	Klein *et al*, Kidney International, *in press*	Cakut	Lower urinary tract obstruction	Normal	34	10
179	AF	Klein *et al*, Kidney International, *in press*	Cakut	Lower urinary tract obstruction	Normal	20	6
180	AF	Klein *et al*, Kidney International, *in press*	Cakut	Lower urinary tract obstruction	Anhydramnios	19	6
181	AF	Klein *et al*, Kidney International, *in press*	Cakut	Lower urinary tract obstruction	Anhydramnios	36	11
182	AF	Klein *et al*, Kidney International, *in press*	Cakut	Lower urinary tract obstruction	n.a	26	8
183	AF	Klein *et al*, Kidney International, *in press*	Cakut	Lower urinary tract obstruction	n.a	27	8
184	AF	Klein *et al*, Kidney International, *in press*	Cakut	Lower urinary tract obstruction	Oligohydramnios	19	6
185	AF	Klein *et al*, Kidney International, *in press*	Cakut	Lower urinary tract obstruction	Oligohydramnios	24	7
186	AF	Klein *et al*, Kidney International, *in press*	Cakut	Lower urinary tract obstruction	Normal	16	5
187	AF	Klein *et al*, Kidney International, *in press*	Cakut	Lower urinary tract obstruction	Oligohydramnios	18	6
188	AF	Klein *et al*, Kidney International, *in press*	Cakut	Lower urinary tract obstruction	Normal	33	10
189	AF	Klein *et al*, Kidney International, *in press*	Cakut	Lower urinary tract obstruction	Oligohydramnios	19	6
190	AF	Klein *et al*, Kidney International, *in press*	Cakut	Lower urinary tract obstruction	n.a	15	5
191	AF	Klein *et al*, Kidney International, *in press*	Cakut	Lower urinary tract obstruction	Oligohydramnios	35	10
192	AF	Klein *et al*, Kidney International, *in press*	Cakut	Lower urinary tract obstruction	Anhydramnios	23	7
193	AF	Klein *et al*, Kidney International, *in press*	Cakut	Lower urinary tract obstruction	n.a	32	10
194	AF	Klein *et al*, Kidney International, *in press*	Cakut	Lower urinary tract obstruction	Oligohydramnios	30	9

Patient code	Analysed biological fluid	Origin of raw data (PMID)	Group	Antenatal etiology (if available)	Amniotic fluid volume "	Gestational age (in weeks of amenorrhea)	Gestational age (in 1/4 trimester)
195	AF	Klein *et al*, Kidney International, *in press*	Cakut	Lower urinary tract obstruction	Oligohydramnios	17	5
196	AF	Klein *et al*, Kidney International, *in press*	Cakut	Lower urinary tract obstruction	Normal	19	6
197	AF	Klein *et al*, Kidney International, *in press*	Cakut	Lower urinary tract obstruction	Oligohydramnios	18	6
198	AF	Klein *et al*, Kidney International, *in press*	Cakut	Lower urinary tract obstruction	Oligohydramnios	24	7
199	AF	Klein *et al*, Kidney International, *in press*	Cakut	Lower urinary tract obstruction	Oligohydramnios	21	6
200	AF	Klein *et al*, Kidney International, *in press*	Cakut	Lower urinary tract obstruction	Oligohydramnios	15	5
201	AF and FU	23946195	Cakut	Lower urinary tract obstruction	Normal	23,5	7
202	AF and FU	23946195	Cakut	Lower urinary tract obstruction	Anhydramnios	16,2	5
203	AF and FU	23946195	Cakut	Lower urinary tract obstruction	Normal	22,4	7
204	AF and FU	23946195	Cakut	Lower urinary tract obstruction	Normal	24	7
205	AF and FU	23946195	Cakut	Lower urinary tract obstruction	Normal	26,3	8
206	AF and FU	23946195	Cakut	Lower urinary tract obstruction	Oligohydramnios	22	7
207	AF and FU	23946195	Cakut	Lower urinary tract obstruction	Normal	26	8
208	AF and FU	23946195	Cakut	Lower urinary tract obstruction	Oligohydramnios	37	11
209	AF and FU	23946195	Cakut	Lower urinary tract obstruction	Normal	34	10
210	AF and FU	23946195	Cakut	Lower urinary tract obstruction	Normal	35,5	11
211	AF and FU	23946195	Cakut	Lower urinary tract obstruction	Normal	14,6	5
212	AF and FU	23946195	Cakut	Lower urinary tract obstruction	Oligohydramnios	31	9
213	AF and FU	23946195	Cakut	Lower urinary tract obstruction	Anhydramnios	33	10
214	AF and FU	23946195	Cakut	Lower urinary tract obstruction	Normal	35,2	10
215	AF and FU	23946195	Cakut	Lower urinary tract obstruction	Normal	23	7
216	AF and FU	23946195	Cakut	Lower urinary tract obstruction	Normal	31,5	10
217	FU	23946195	Cakut	Lower urinary tract obstruction	Normal	15	5
218	FU	23946195	Cakut	Lower urinary tract obstruction	Normal	17	5
219	FU	23946195	Cakut	Lower urinary tract obstruction	Normal	22	7
220	FU	23946195	Cakut	Lower urinary tract obstruction	Normal	23	7
221	FU	23946195	Cakut	Lower urinary tract obstruction	Normal	23	7
222	FU	23946195	Cakut	Lower urinary tract obstruction	Normal	23	7
223	FU	23946195	Cakut	Lower urinary tract obstruction	Normal	24	7
224	FU	23946195	Cakut	Lower urinary tract obstruction	Normal	24	7
225	FU	23946195	Cakut	Lower urinary tract obstruction	Normal	24	7
226	FU	23946195	Cakut	Lower urinary tract obstruction	Normal	24	7
227	FU	23946195	Cakut	Lower urinary tract obstruction	Normal	25	8
228	FU	23946195	Cakut	Lower urinary tract obstruction	Normal	25	8
229	FU	23946195	Cakut	Lower urinary tract obstruction	Normal	25	8
230	FU	23946195	Cakut	Lower urinary tract obstruction	Normal	26	8
231	FU	23946195	Cakut	Lower urinary tract obstruction	Normal	26	8
232	FU	23946195	Cakut	Lower urinary tract obstruction	Normal	26	8
233	FU	23946195	Cakut	Lower urinary tract obstruction	Normal	26	8
234	FU	23946195	Cakut	Lower urinary tract obstruction	Normal	27	8
235	FU	23946195	Cakut	Lower urinary tract obstruction	Normal	27	8
236	FU	23946195	Cakut	Lower urinary tract obstruction	Normal	28	8
237	FU	23946195	Cakut	Lower urinary tract obstruction	Normal	29	9
238	FU	23946195	Cakut	Lower urinary tract obstruction	Normal	29	9
239	FU	23946195	Cakut	Lower urinary tract obstruction	Normal	29	9
240	FU	23946195	Cakut	Lower urinary tract obstruction	Normal	32	10
241	FU	23946195	Cakut	Lower urinary tract obstruction	Normal	32	10
242	FU	23946195	Cakut	Lower urinary tract obstruction	Normal	33	10
243	FU	23946195	Cakut	Lower urinary tract obstruction	Normal	34	10
244	FU	23946195	Cakut	Lower urinary tract obstruction	Normal	34	10
245	FU	23946195	Cakut	Lower urinary tract obstruction	Normal	34	10
246	FU	23946195	Cakut	Lower urinary tract obstruction	Normal	35	10
247	FU	23946195	Cakut	Lower urinary tract obstruction	Normal	35	10
248	FU	23946195	Cakut	Lower urinary tract obstruction	Normal	36	11
249	FU	23946195	Cakut	Lower urinary tract obstruction	Oligohydramnios	17	5
250	FU	23946195	Cakut	Lower urinary tract obstruction	Oligohydramnios	17	5
251	FU	23946195	Cakut	Lower urinary tract obstruction	Oligohydramnios	18	6
252	FU	23946195	Cakut	Lower urinary tract obstruction	Oligohydramnios	22	7
253	FU	23946195	Cakut	Lower urinary tract obstruction	Oligohydramnios	23	7
254	FU	23946195	Cakut	Lower urinary tract obstruction	Oligohydramnios	27	8
255	FU	23946195	Cakut	Lower urinary tract obstruction	Oligohydramnios	27	8
256	FU	23946195	Cakut	Lower urinary tract obstruction	Oligohydramnios	28	8
257	FU	23946195	Cakut	Lower urinary tract obstruction	Oligohydramnios	29	9
258	FU	23946195	Cakut	Lower urinary tract obstruction	Oligohydramnios	30	9
259	FU	23946195	Cakut	Lower urinary tract obstruction	Oligohydramnios	31	9
260	FU	23946195	Cakut	Lower urinary tract obstruction	Oligohydramnios	31	9
261	FU	23946195	Cakut	Lower urinary tract obstruction	Oligohydramnios	31	9
262	FU	23946195	Cakut	Lower urinary tract obstruction	Oligohydramnios	33	10
263	FU	23946195	Cakut	Lower urinary tract obstruction	Oligohydramnios	33	10
264	FU	23946195	Cakut	Lower urinary tract obstruction	Oligohydramnios	33	10
265	FU	23946195	Cakut	Lower urinary tract obstruction	Oligohydramnios	33	10
266	FU	23946195	Cakut	Lower urinary tract obstruction	Oligohydramnios	34	10
267	FU	23946195	Cakut	Lower urinary tract obstruction	Oligohydramnios	35	10
268	FU	23946195	Cakut	Lower urinary tract obstruction	Oligohydramnios	37	11
269	FU	23946195	Cakut	Lower urinary tract obstruction	Anhydramnios	16	5
270	FU	23946195	Cakut	Lower urinary tract obstruction	Anhydramnios	19	6
271	FU	23946195	Cakut	Lower urinary tract obstruction	Anhydramnios	20	6
272	FU	23946195	Cakut	Lower urinary tract obstruction	Anhydramnios	22	7
273	FU	23946195	Cakut	Lower urinary tract obstruction	Anhydramnios	30	9
274	FU	23946195	Cakut	Lower urinary tract obstruction	Anhydramnios	32	10
275	FU	23946195	Cakut	Lower urinary tract obstruction	Anhydramnios	32	10
276	FU	23946195	Cakut	Lower urinary tract obstruction	Anhydramnios	32	10
277	FU	23946195	Cakut	Lower urinary tract obstruction	Anhydramnios	33	10
278	FU	23946195	Cakut	Lower urinary tract obstruction	Anhydramnios	33	10
279	FU	23946195	Cakut	Lower urinary tract obstruction	Anhydramnios	33	10
280	FU	23946195	Cakut	Lower urinary tract obstruction	Anhydramnios	34	10

* AF: amniotic fluid.

** FU: fetal urine.

" Oligohydramnios/anhydramnios: amniotic fluid volume was estimated using the amniotic fluid index (AFI) which is defined as the sum of the largest vertical fluid pocket in each of the four quadrants of the maternal abdomen. An AFI of <5 indicated the presence of oligohydramnios, an AFI of ~0 was considered as absence of amniotic fluid (anhydramnios); n.a, not available.

Supplementary Table 2: List of correlated common peptides in AF and FU

Peptide ID	Correlation coefficient	pvalue	Peptide sequence	Original protein name	Original protein ID
48151	0.839	0	n.a	n.a	n.a
5116	0.881	0	pGDRGEpGPpGP	Collagen alpha-1(I) chain	P02452
18627	0.824	0.001	NSGEpGApGSKGDTGAKGEp	Collagen alpha-1(I) chain	P02452
4113	0.797	0.002	n.a	n.a	n.a
20861	0.818	0.002	n.a	n.a	n.a
28320	0.791	0.002	n.a	n.a	n.a
957	0.867	0.003	n.a	n.a	n.a
18837	1	0.003	GppGPPGRDGEDGpTGPpGP	Collagen alpha-2(I) chain	P08123
17300	0.703	0.003	n.a	n.a	n.a
62216	0.769	0.003	n.a	n.a	n.a
29445	0.783	0.004	n.a	n.a	n.a
6968	0.867	0.005	TGPpGDpGDRGpP	Collagen alpha-1(XI) chain	P12107
79671	0.704	0.005	n.a	n.a	n.a
51134	0.686	0.006	n.a	n.a	n.a
82931	0.929	0.007	n.a	n.a	n.a
49762	0.773	0.008	n.a	n.a	n.a
10250	0.806	0.008	HSHEDMLVVDPK	Osteopontin	P10451
54348	0.833	0.008	n.a	n.a	n.a
55286	0.661	0.009	n.a	n.a	n.a
10640	0.657	0.01	SVPGPpGPpGPpGPpG	Collagen alpha-1(XVIII) chain	P39060
31138	0.703	0.01	n.a	n.a	n.a
16197	0.65	0.011	PpGEAGKpGEQGVpGDLG	Collagen alpha-1(I) chain	P02452
74166	0.857	0.011	n.a	n.a	n.a
9946	0.626	0.011	n.a	n.a	n.a
81073	0.8	0.014	n.a	n.a	n.a
10169	0.625	0.015	n.a	n.a	n.a
26921	0.727	0.015	n.a	n.a	n.a
34490	0.603	0.015	n.a	n.a	n.a
1318012	0.644	0.015	n.a	n.a	n.a
55365	0.692	0.016	n.a	n.a	n.a
29894	0.64	0.016	AGPpGApGAPGAPGpVGPAGKSGDRGETGP	Collagen alpha-1(I) chain	P02452
34039	1	0.017	n.a	n.a	n.a
52885	1	0.017	n.a	n.a	n.a
20873	1	0.017	n.a	n.a	n.a
51534	0.943	0.017	n.a	n.a	n.a
6336	0.943	0.017	n.a	n.a	n.a
18054	0.631	0.018	n.a	n.a	n.a
46179	0.81	0.022	[illegible]RGEAGAAGpAGpAGPRGSPGERGEVGpAGPNGFAGpAG	Collagen alpha-2(I) chain	P08123
429	0.664	0.022	n.a	n.a	n.a
5694	0.857	0.024	n.a	n.a	n.a
63055	0.857	0.024	n.a	n.a	n.a
60149	0.6	0.026	n.a	n.a	n.a
21981	0.579	0.026	GpPGEAGKpGEQGVPGDLGApGP	Collagen alpha-1(I) chain	P02452
7212	0.596	0.028	n.a	n.a	n.a
51209	0.786	0.028	n.a	n.a	n.a
1323464	0.615	0.029	n.a	n.a	n.a
39425	0.664	0.031	n.a	n.a	n.a
4586	0.697	0.031	n.a	n.a	n.a
64801	0.629	0.032	n.a	n.a	n.a
33991	0.886	0.033	GGPpGPKGNSGEpGApGSKGDTGAKGEpGPVG	Collagen alpha-1(I) chain	P02452
32038	0.886	0.033	GPpGPpGYGSQGIKGEQGpQGFPGpKGT	Collagen alpha-1(XXVIII) chain	Q2UY09
33402	0.886	0.033	GPQGPpGPSGEEGKRGPNGEAGSAGPpGPpG	Collagen alpha-2(I) chain	P08123
53761	0.886	0.033	n.a	n.a	n.a
38764	0.886	0.033	n.a	n.a	n.a
60341	0.886	0.033	n.a	n.a	n.a
62323	0.886	0.033	n.a	n.a	n.a
35226	0.557	0.034	RGPpGPpGKNGDDGEAGKPGRPGERGppGP	Collagen alpha-1(I) chain	P02452
80518	0.821	0.034	n.a	n.a	n.a
69605	0.661	0.044	n.a	n.a	n.a
10307	0.512	0.045	n.a	n.a	n.a
81033	0.594	0.046	n.a	n.a	n.a
17264	0.738	0.046	GPpGEAGKpGEQGVpGDLG	Collagen alpha-1(I) chain	P02452
62435	0.738	0.046	n.a	n.a	n.a
51675	0.738	0.046	n.a	n.a	n.a
10072	0.786	0.048	SpGAPGAPGHpGPPGp	Collagen alpha-1(III) chain	P02461
70401	0.786	0.048	n.a	n.a	n.a
4358	0.648	0.049	n.a	n.a	n.a

n.a, not available.

Supplementary Table 3: Subset of 30 fetuses with PUV used for peptide based classifiers									
**Patient code**	**Analysed biological fluid**	**Cohort**	**Clinical postnatal outcome at 2 years ᵞ**	**AF7cPUV score based prediction**			**(FU)12PUV score based prediction**		
				**Score**	**Predicted postnatal outcome**		**Score**	**Predicted postnatal outcome**	
173	AF	Discovery	ESRD	1					
174	AF	Discovery	noESRD	-1.0005					
175	AF	Discovery	noESRD	-0.9998					
178	AF	Discovery	noESRD	-0.9998					
183	AF	Discovery	ESRD	1					
186	AF	Discovery	noESRD	-1.0002					
187	AF	Discovery	ESRD	0.7446					
188	AF	Discovery	noESRD	-0.9995					
189	AF	Discovery	noESRD	0.3228					
191	AF	Discovery	noESRD	-1.0002					
192	AF	Discovery	ESRD	1.0002					
193	AF	Discovery	noESRD	-1.0003					
196	AF	Discovery	ESRD	1.0001					
198	AF	Discovery	ESRD	0.8644					
201	AF and FU	Validation	noESRD	-0.8164	noESRD	**correct**	-1.544	noESRD	**correct**
202	AF and FU	Validation	ESRD	0.0852	ESRD	**correct**	0.129	ESRD	**correct**
203	AF and FU	Validation	noESRD	-0.6562	noESRD	**correct**	-1.431	noESRD	**correct**
204	AF and FU	Validation	noESRD	-0.7396	noESRD	**correct**	-2.129	noESRD	**correct**
205	AF and FU	Validation	noESRD	-0.7443	noESRD	**correct**	-0.331	noESRD	**correct**
206	AF and FU	Validation	ESRD	1.0097	ESRD	**correct**	-0.697	noESRD	**mispredicted**
207	AF and FU	Validation	ESRD	0.8377	ESRD	**correct**	1.703	ESRD	**correct**
208	AF and FU	Validation	noESRD	-0.8723	noESRD	**correct**	-1.432	noESRD	**correct**
209	AF and FU	Validation	noESRD	0.4776	ESRD	**mispredicted**	-1.187	noESRD	**correct**
210	AF and FU	Validation	ESRD	0.586	ESRD	**correct**	1.25	ESRD	**correct**
211	AF and FU	Validation	ESRD	0.7223	ESRD	**correct**	0.161	ESRD	**correct**
212	AF and FU	Validation	noESRD	-0.7229	noESRD	**correct**	-2.66	noESRD	**correct**
213	AF and FU	Validation	ESRD	0.0633	ESRD	**correct**	1.177	ESRD	**correct**
214	AF and FU	Validation	noESRD	-0.6366	noESRD	**correct**	-0.58	noESRD	**correct**
215	AF and FU	Validation	ESRD	0.7748	ESRD	**correct**	1.976	ESRD	**correct**
216	AF and FU	Validation	ESRD	0.6491	ESRD	**correct**	0.001	ESRD	**correct**

* AF: amniotic fluid.

** FU: fetal urine.

ᵞClinical postnatal outcome: ESRD, end-stage renal disease (death in the neonatal period due to ESRD or termination of pregnancy due to the severity of renal lesions); noESRD, glomerular filtration rate >15 ml/min/1.73m^2.

Supplementary Table 4: List of ExPASY retrieved peptidases cleaving FU specific fragments of collagen alpha-1(I) chain

Peptide ID	Original protein name	Original protein ID	Start position in original protein	Stop position in original protein	Peptidase name (cut number in peptide)	Cleavage sites in sequence of peptide*
5802	Collagen alpha-1(I) chain	P02452	107	119	Trypsin (1)	Tryps<br>TTGVEGPKGDTGP
20731	Collagen alpha-1(I) chain	P02452	317	340	Arg-C proteinase (1)<br>Trypsin (1)	ArgC, Tryps<br>GPAGARGNDGATGAAGppGPTGPA
33196	Collagen alpha-1(I) chain	P02452	372	404	Trypsin (1)	Tryps<br>EPGPpGPAGAAGPAGNpGADGQpGAKGANGApG
30468	Collagen alpha-1(I) chain	P02452	418	447	Trypsin (2)	Tryps Tryps<br>SGPQGPGGPpGPKGNSGEpGApGSKGDTGA
21461	Collagen alpha-1(I) chain	P02452	433	455	Trypsin (2)	Tryps Tryps<br>SGEpGApGSKGDTGAKGEpGPVG
5423	Collagen alpha-1(I) chain	P02452	546	558	Trypsin (1)	Tryps<br>SpGPDGKTGPpGP
220	Collagen alpha-1(I) chain	P02452	550	558	Trypsin (1)	Tryps<br>DGKTGPpGP
23166	Collagen alpha-1(I) chain	P02452	586	609	Arg-C proteinase (1)<br>Trypsin (3)	Tryps<br>ArgC, Tryps<br>Tryps<br>KGAAGEpGKAGERGVpGPpGAVGP
17468	Collagen alpha-1(I) chain	P02452	587	606	Arg-C proteinase (1)<br>Trypsin (2)	ArgC, Tryps<br>Tryps<br>GAAGEpGKAGERGVpGPpGA
13493	Collagen alpha-1(I) chain	P02452	707	725	Trypsin (1)	Tryps<br>GAKGDAGApGApGSQGApG
45688	Collagen alpha-1(I) chain	P02452	726	764	Arg-C proteinase (3)<br>Chymotrypsin (1)<br>Pepsin (2)<br>Trypsin (6)	ArgC, Tryps<br>Tryps<br>ArgC, Tryps Tryps<br>Tryps<br>Peps<br>ArgC, Tryps<br>Chym, Peps<br>LQGMpGERGAAGLPGpKGDRGDAGpKGADGSpGKDGVRG
43075	Collagen alpha-1(I) chain	P02452	809	849	Chymotrypsin (1)<br>Pepsin (2)<br>Trypsin (2)	Chym, Peps Tryps Tryps<br>Peps<br>GpAGFAGpPGADGQPGAKGEPGDAGAKGDAGPpGpAGPAGP
28872	Collagen alpha-1(I) chain	P02452	815	843	Trypsin (2)	Tryps Tryps<br>GPpGADGQPGAKGEPGDAGAKGDAGPpGP
11703	Collagen alpha-1(I) chain	P02452	820	835	Trypsin (2)	Tryps Tryps<br>DGQpGAKGEpGDAGAK
5397	Collagen alpha-1(I) chain	P02452	940	953	-	
29488	Collagen alpha-1(I) chain	P02452	1007	1034	Arg-C proteinase (2)<br>Trypsin (3)	Tryps<br>ArgC, Tryps<br>ArgC, Tryps<br>GPpGESGREGApGAEGSpGRDGSpGAKG
4403	Collagen alpha-1(I) chain	P02452	1008	1019	Arg-C proteinase (1)<br>Trypsin (1)	ArgC, Tryps<br>PpGESGREGApG
36044	Collagen alpha-1(I) chain	P02452	1010	1041	Arg-C proteinase (3)<br>Trypsin (4)	ArgC, Tryps<br>Tryps<br>ArgC, Tryps<br>ArgC, Tryps<br>GESGREGAPGAEGSpGRDGSPGAKGDRGETGp
19915	Collagen alpha-1(I) chain	P02452	1050	1071	Arg-C proteinase (1)<br>Trypsin (2)	ArgC, Tryps<br>Tryps<br>ApGAPGPVGPAGKSGDRGETGP
45508	Collagen alpha-1(I) chain	P02452	1095	1131	Arg-C proteinase (2)<br>Chymotrypsin (3)<br>Pepsin (2)<br>Trypsin (4)	Chym, Peps<br>Peps<br>Chym<br>ArgC, Tryps<br>Chym<br>Tryps<br>ArgC, Tryps<br>Tryps<br>DKGETGEQGDRGIKGHRGFSGLQGppGPpGSPGEQGP
10802	Collagen alpha-1(I) chain	P02452	1177	1192	-	
14399	Collagen alpha-1(I) chain	P02452	1177	1195	-	

We used ExPASy (https://web.expasy.org/peptide_cutter/) to identify possible peptidases able to induce degradation of 22 fragments of Collagen alpha-1(I) chain found exlusively in FU. Of note, simulation was performed only with peptidases displaying a human annotation in UniProt database. *p, hydroxyproline

Supplementary Table 5: List of PROTEASIX retrieved proteases generating AF specific peptides

Protease ID	Protease name	Detection in AF (PMID)	Cleavage sequence	Protease substrat ID	Protease substrat name	N or C-terminus	Generated peptide ID	Generated peptide sequence*	Generated peptide position in protease substrat
ADAMTS2 (O95450; ATS2_HUMAN)	A disintegrin and metalloproteinase with thrombospondin motifs 2	29684686, 29781159	NFAAQYDG	P08123	Collagen alpha-2(I) chain	C-terminus	18852	TGPpGPpGPpGPpGLGGNFAA	59 - 79
ADAMTS2 (O95450; ATS2_HUMAN)	A disintegrin and metalloproteinase with thrombospondin motifs 2 followed by exopeptidase (+3AA)		NFAAQYDG	P08123	Collagen alpha-2(I) chain	C-terminus	13797	TGPpGPpGPpGPpGLGGN	59 - 76
ADAMTS5 (Q9UNA0; ATS5_HUMAN)	A disintegrin and metalloproteinase with thrombospondin motifs 5 followed by exopeptidase (+1AA)	29684686, 29781159, 30730845, 30623540	PGPQGHAG	P02461	Collagen alpha-1(III) chain	C-terminus	29197	SpGAKGEVGPAGSpGSNGApGQRGEpGP	346 - 373
BMP1 (P13497; BMP1_HUMAN)	Bone morphogenetic protein 1	29684686, 29781159	SGGNDAPG	P98160	Basement membrane-specific heparan sulfate proteoglycan core protein	N-terminus	30383	DAPGQYGAYFHDDGFLAFPGHVFS	4197 - 4220
CAPN1 (P07384; CAN1_HUMAN)	Calpain-1 catalytic subunit	31704631, 29684686, 29781159	NVKAAWGK	P69905	Hemoglobin subunit alpha	C-terminus	6413	VLSPADKTNVKA	2 - 13
CAPN1 (P07384; CAN1_HUMAN)	Calpain-1 catalytic subunit followed by exopeptidase (+1AA)		VKAAWGKV	P69905	Hemoglobin subunit alpha	C-terminus	6413	VLSPADKTNVKA	2 - 13
CAPN1 (P07384; CAN1_HUMAN)	Calpain-1 catalytic subunit followed by exopeptidase (+2AA)		KAAWGKVG	P69905	Hemoglobin subunit alpha	C-terminus	6413	VLSPADKTNVKA	2 - 13
CAPN1 (P07384; CAN1_HUMAN)	Calpain-1 catalytic subunit followed by exopeptidase (+3AA)		AAWGKVGA	P69905	Hemoglobin subunit alpha	C-terminus	6413	VLSPADKTNVKA	2 - 13
CFI (P05156; CFAI_HUMAN)	Complement factor I	29684686, 17373841, 29781159	SLLRSEET	P01024	Complement C3	N-terminus	11411	SEETKENEGFTVT	1321 - 1333
CFI (P05156; CFAI_HUMAN)	Complement factor I		SLLRSEET	P01024	Complement C3	C-terminus	5723	IHWESASLLR	1311 - 1320
CFI (P05156; CFAI_HUMAN)	Complement factor I followed by exopeptidase (+1AA)		SLLRSEET	P01024	Complement C3	C-terminus	1189	HWESASLL	1312 - 1319
CTSD (P07339; CATD_HUMAN)	Cathepsin D followed by exopeptidase (+1AA)		TQRFFESF	F8W6P5	Hemoglobin subunit beta	N-terminus	18674	ESFGDLSTPDAVMGNPKV	44 - 61
CTSD (P07339; CATD_HUMAN)	Cathepsin D followed by exopeptidase (+2AA)		QSRLYKRK	P10451	Osteopontin	C-terminus	45848	LNAPSDWDSRGKDSYETSQLDDQSAETHSHKQS	211 - 243
CTSD (P07339; CATD_HUMAN)	Cathepsin D followed by exopeptidase (+2AA)		QSRLYKRK	P10451	Osteopontin	C-terminus	25079	SYETSQLDDQSAETHSHKQS	224 - 243
CTSK (P43235; CATK_HUMAN)	Cathepsin K followed by exopeptidase (+2AA)		DFSFLPQP	P02452	Collagen alpha-1(I) chain	C-terminus	21532	VGPpGPpGPpGPpGPPSAGFDF	1177 - 1198
CTSK (P43235; CATK_HUMAN)	Cathepsin K		MGPRGPPG	P02458	Collagen alpha-1(II) chain	N-terminus	31783	GPpGPpGKPGDDGEAGKpGKAGERGpPGP	243 - 271
CTSK (P43235; CATK_HUMAN)	Cathepsin K		GPPGAPGP	P02461	Collagen alpha-1(III) chain	C-terminus	15150	SEGSPGHpGQPGPpGPpG	1174 - 1191
CTSL (P07711; CATL1_HUMAN)	Cathepsin L1	29684686, 29781159	GPPGAPGP	P02461	Collagen alpha-1(III) chain	C-terminus	15150	SEGSPGHpGQPGPpGPpG	1174 - 1191
CTSS (P25774; CATS_HUMAN)	Cathepsin S	29684686, 29781159	KLKKTETQ	P62328	Thymosin beta-4	N-terminus	31862	TETQEKNPLPSKETIEQEKQAGES	21 - 44
ECE2 (O60344; ECE2_HUMAN)	Endothelin-converting enzyme 2		VLGALLRV	Q9UHG2	ProSAAS	C-terminus	13891	DHDVGSELPPEGVLGA	223 - 238
ECE2 (O60344; ECE2_HUMAN)	Endothelin-converting enzyme 2		GALLRVKR	Q9UHG2	ProSAAS	C-terminus	18008	DHDVGSELPPEGVLGALL	223 - 240
ECE2 (O60344; ECE2_HUMAN)	Endothelin-converting enzyme 2 followed by exopeptidase (+1AA)		ALLRVKRL	Q9UHG2	ProSAAS	C-terminus	18008	DHDVGSELPPEGVLGALL	223 - 240
ECE2 (O60344; ECE2_HUMAN)	Endothelin-converting enzyme 2 followed by exopeptidase (+2AA)		GALLRVKR	Q9UHG2	ProSAAS	C-terminus	13891	DHDVGSELPPEGVLGA	223 - 238
ECE2 (O60344; ECE2_HUMAN)	Endothelin-converting enzyme 2 followed by exopeptidase (+3AA)		ALLRVKRL	Q9UHG2	ProSAAS	C-terminus	13891	DHDVGSELPPEGVLGA	223 - 238
ELANE (P08246; ELNE_HUMAN)	Neutrophil elastase followed by exopeptidase (+2AA)	29781159, 28167848, 27628770, 27216361, 2	HVLLAAPP	C9JV77	Alpha-2-HS-glycoprotein	C-terminus	5005	LPPAGSPPDSHV	289 - 300
F2 (P00734; THRB_HUMAN)	Prothrombin followed by exopeptidase (+1AA)	31704631, 29684686, 17373841, 29781159	KQSRLYKR	P10451	Osteopontin	C-terminus	45848	LNAPSDWDSRGKDSYETSQLDDQSAETHSHKQS	211 - 243
F2 (P00734; THRB_HUMAN)	Prothrombin followed by exopeptidase (+1AA)		KQSRLYKR	P10451	Osteopontin	C-terminus	25079	SYETSQLDDQSAETHSHKQS	224 - 243
KLK14 (Q9P0G3; KLK14_HUMAN)	Kallikrein-14 followed by exopeptidase (+1AA)	29781159, 14695222	DGFRHRHP	P02671	Fibrinogen alpha chain	N-terminus	14428	RHPDEAAFFDTASTG	512 - 526
MASP1 (P48740; MASP1_HUMAN)	Mannan-binding lectin serine protease 1 followed by exopeptidase (+1AA)	29684686	SLGRKIQI	P06681	Complement C2	C-terminus	19204	SHMLGATNPTQKTKESLG	225 - 242
MASP1 (P48740; MASP1_HUMAN)	Mannan-binding lectin serine protease 1 followed by exopeptidase (+1AA)		SLGRKIQI	P06681	Complement C2	C-terminus	15511	MLGATNPTQKTKESLG	227 - 242
MASP2 (O00187; MASP2_HUMAN)	Mannan-binding lectin serine protease 2 followed by exopeptidase (+1AA)	31704631, 29684686, 29781159	SLGRKIQI	P06681	Complement C2	C-terminus	19204	SHMLGATNPTQKTKESLG	225 - 242
MASP2 (O00187; MASP2_HUMAN)	Mannan-binding lectin serine protease 2 followed by exopeptidase (+1AA)		SLGRKIQI	P06681	Complement C2	C-terminus	15511	MLGATNPTQKTKESLG	227 - 242
MMP12 (P39900; MMP12_HUMAN)	Macrophage metalloelastase followed by exopeptidase (+3AA)	29684686, 29781159	ALEDLRQG	P02647	Apolipoprotein A-I	C-terminus	13734	ATEHLSTLSEKAKPA	220 - 234
MMP13 (P45452; MMP13_HUMAN)	Collagenase 3	15085886	GPQGLQGL	P02461	Collagen alpha-1(III) chain	C-terminus	12220	TGPGGDKGDTGPpGPQG	623 - 639
MMP13 (P45452; MMP13_HUMAN)	Collagenase 3 followed by exopeptidase (+2AA)		GPPGAIGP	P02461	Collagen alpha-1(III) chain	C-terminus	21392	SpGYQGPPGEPGQAGpSGppGP	196 - 217
MMP2 (P08253; MMP2_HUMAN)	72 kDa type IV collagenase followed by exopeptidase (+3AA)	29684686, 29781159, 23796141, 26076029, 2	GPPGLAGP	P02452	Collagen alpha-1(I) chain	N-terminus	4402	PpGESGREGApG	1008 - 1019
MMP2 (P08253; MMP2_HUMAN)	72 kDa type IV collagenase		GPRGRSGE	P02458	Collagen alpha-1(II) chain	N-terminus	24859	RSGETGPAGppGNPGPpGPPGPpGP	1192 - 1216
MMP2 (P08253; MMP2_HUMAN)	72 kDa type IV collagenase		SKLKKTET	P62328	Thymosin beta-4	N-terminus	33930	KTETQEKNPLPSKETIEQEKQAGES	20 - 44
MMP2 (P08253; MMP2_HUMAN)	72 kDa type IV collagenase followed by exopeptidase (+1AA)		SKLKKTET	P62328	Thymosin beta-4	N-terminus	31862	TETQEKNPLPSKETIEQEKQAGES	21 - 44
MMP20 (O60882; MMP20_HUMAN)	Matrix metalloproteinase-20		GPQGLQGL	P02461	Collagen alpha-1(III) chain	C-terminus	12220	TGPGGDKGDTGPpGPQG	623 - 639
MMP25 (Q9NPA2; MMP25_HUMAN)	Matrix metalloproteinase-25 followed by exopeptidase (+2AA)		PPGLAGPP	P02452	Collagen alpha-1(I) chain	N-terminus	4402	PpGESGREGApG	1008 - 1019
MMP25 (Q9NPA2; MMP25_HUMAN)	Matrix metalloproteinase-25 followed by exopeptidase (+3AA)		GPPGLAGP	P02452	Collagen alpha-1(I) chain	N-terminus	4402	PpGESGREGApG	1008 - 1019
MMP25 (Q9NPA2; MMP25_HUMAN)	Matrix metalloproteinase-25 followed by exopeptidase (+2AA)		PGAPGAPG	P02461	Collagen alpha-1(III) chain	C-terminus	45588	GLAGTAGEpGRDGNpGSDGLpGRDGSpGGKGDRGENGSPG	1002 - 1041
MMP25 (Q9NPA2; MMP25_HUMAN)	Matrix metalloproteinase-25 followed by exopeptidase (+3AA)		AGPQGPPG	P08123	Collagen alpha-2(I) chain	C-terminus	22385	LVGEpGPAGSKGESGNKGEpGSA	344 - 366
MMP26 (Q9NRE1; MMP26_HUMAN)	Matrix metalloproteinase-26		KPEGIDSR	P04004	Vitronectin	C-terminus	8765	EAPAPEVGASKPEG	121 - 134
MMP3 (P08254; MMP3_HUMAN)	Stromelysin-1	29781159, 26076029, 25856763	GPRGRSGE	P02458	Collagen alpha-1(II) chain	N-terminus	24859	RSGETGPAGppGNPGPpGPPGPpGP	1192 - 1216
MMP3 (P08254; MMP3_HUMAN)	Stromelysin-1		VAQDLNAP	P10451	Osteopontin	N-terminus	45848	LNAPSDWDSRGKDSYETSQLDDQSAETHSHKQS	211 - 243
MMP7 (P09237; MMP7_HUMAN)	Matrilysin followed by exopeptidase (+1AA)	31704631, 29781159	HLSTLSEK	P02647	Apolipoprotein A-I	N-terminus	5496	SEKAKPALEDL	228 - 238
MMP7 (P09237; MMP7_HUMAN)	Matrilysin followed by exopeptidase (+3AA)		ALEDLRQG	P02647	Apolipoprotein A-I	C-terminus	13734	ATEHLSTLSEKAKPA	220 - 234
MMP7 (P09237; MMP7_HUMAN)	Matrilysin		VAQDLNAP	P10451	Osteopontin	N-terminus	45848	LNAPSDWDSRGKDSYETSQLDDQSAETHSHKQS	211 - 243
MMP8 (P22894; MMP8_HUMAN)	Neutrophil collagenase	29781159, 23796141, 26076029, 25856763, 2	GPRGRSGE	P02458	Collagen alpha-1(II) chain	N-terminus	24859	RSGETGPAGppGNPGPpGPPGPpGP	1192 - 1216
MMP9 (P14780; MMP9_HUMAN)	Matrix metalloproteinase-9 followed by exopeptidase (+3AA)	29684686, 29781159, 26076029, 25856763, 2	GPPGLAGP	P02452	Collagen alpha-1(I) chain	N-terminus	4402	PpGESGREGApG	1008 - 1019
MMP9 (P14780; MMP9_HUMAN)	Matrix metalloproteinase-9 followed by exopeptidase (+2AA)		GPQGARGF	P02458	Collagen alpha-1(II) chain	C-terminus	31783	GPpGPpGKPGDDGEAGKpGKAGERGpPGP	243 - 271
MMP9 (P14780; MMP9_HUMAN)	Matrix metalloproteinase-9		GPQGLQGL	P02461	Collagen alpha-1(III) chain	C-terminus	12220	TGPGGDKGDTGPpGPQG	623 - 639
MMP9 (P14780; MMP9_HUMAN)	Matrix metalloproteinase-9 followed by exopeptidase (+2AA)		GPPGAIGP	P02461	Collagen alpha-1(III) chain	C-terminus	21392	SpGYQGPPGEPGQAGpSGppGP	196 - 217
NAPSA (O96009; NAPSA_HUMAN)	Napsin-A followed by exopeptidase (+1AA)	31704631, 29781159	KLVLPVLP	H0Y7V6	Pulmonary surfactant-associated protein B	C-terminus	14095	LRDPLPDPLLDKLV	161 - 174
NAPSA (O96009; NAPSA_HUMAN)	Napsin-A followed by exopeptidase (+2AA)		KLVLPVLP	H0Y7V6	Pulmonary surfactant-associated protein B	C-terminus	6238	DPLPDPLLDKL	163 - 173
NAPSA (O96009; NAPSA_HUMAN)	Napsin-A followed by exopeptidase (+3AA)		VLPVLPGA	H0Y7V6	Pulmonary surfactant-associated protein B	C-terminus	14095	LRDPLPDPLLDKLV	161 - 174
PLG (P00747; PLMN_HUMAN)	Plasminogen followed by exopeptidase (+2AA)	17373841, 16900778, 17495049, 29781159, 1	GGYRARPA	P02675	Fibrinogen beta chain	C-terminus	23126	DKKREEAPSLRPAPPPISGGG	50 - 70
PLG (P00747; PLMN_HUMAN)	Plasminogen followed by exopeptidase (+3AA)		KHLKFRIS	P10451	Osteopontin	N-terminus	7149	SHELDSASSEVN	303 - 314

Given the broad substrate specificity of most proteases, one protease could be assigned to different peptide sequences (i.e predicted cleavage by matrix metalloproteinase (MMP)-25 of alpha-1(I), alpha-1(III) or alpha-2(I) chains of collagen) or, conversely, one peptide sequence could be cleaved by different proteases (i.e predicted cleavage of osteopontin [211–243] fragment by cathepsin D, prothrombin, stromelysin-1 (MMP-3) or matrilysin (MMP-7)). *p, hydroxyproline
